# Diminazen Aceturate Protects Pulmonary Ischemia-Reperfusion Injury via Inhibition of ADAM17-Mediated Angiotensin-Converting Enzyme 2 Shedding

**DOI:** 10.3389/fphar.2021.713632

**Published:** 2021-10-12

**Authors:** Li-Fang Wang, Yang-Yang Sun, Qian Pan, Yi-Qing Yin, Xiao-Ming Tian, Yue Liu, Tegeleqi Bu, Qingy Zhang, Yong-An Wang, Jing Zhao, Yuan Luo

**Affiliations:** ^1^ China-Japan Friendship Hospital, Beijing, China; ^2^ Institute of Pharmacology and Toxicology, Academy of Military Medical Sciences (AMMS), Beijing, China

**Keywords:** pulmonary reperfusion injury, angiotensin-converting enzyme 2, ACE2 shedding, renin-angiotensin system, diminazen aceturate

## Abstract

Lung ischemia-reperfusion (IR) injury is induced by pulmonary artery occlusion and reperfusion. Lung IR injury commonly happens after weaning from extracorporeal circulation, lung transplantation, and pulmonary thromboendarterectomy; it is a lethal perioperative complication. A definite therapeutic intervention remains to be determined. It is known that the enzyme activity of angiotensin-converting enzyme 2 (ACE2) is critical in maintaining pulmonary vascular tone and epithelial integrity. In a noxious environment to the lungs, inactivation of ACE2 is mainly due to a disintegrin and metalloprotease 17 (ADAM17) protein-mediated ACE2 shedding. Thus, we assumed that protection of local ACE2 in the lung against ADAM17-mediated shedding would be a therapeutic target for lung IR injury. In this study, we established both *in vivo* and *in vitro* models to demonstrate that the damage degree of lung IR injury depends on the loss of ACE2 and ACE2 enzyme dysfunction in lung tissue. Treatment with ACE2 protectant diminazen aceturate (DIZE) maintained higher ACE2 enzyme activity and reduced angiotensin II, angiotensin type 1 receptor, and ADAM17 levels in the lung tissue. Concurrently, DIZE-inhibited oxidative stress and nitrosative stress via p38MAPK and NF-κB pathways consequently reduced release of pro-inflammatory cytokines such as TNF-α, IL-6, and IL-1β. The underlying molecular mechanism of DIZE contributed to its protective effect against lung IR injury and resulted in the improvement of oxygenation index and ameliorating pulmonary pathological damage. We concluded that DIZE protects the lungs from IR injury via inhibition of ADAM17-mediated ACE2 shedding.

**Graphical Abstract:**
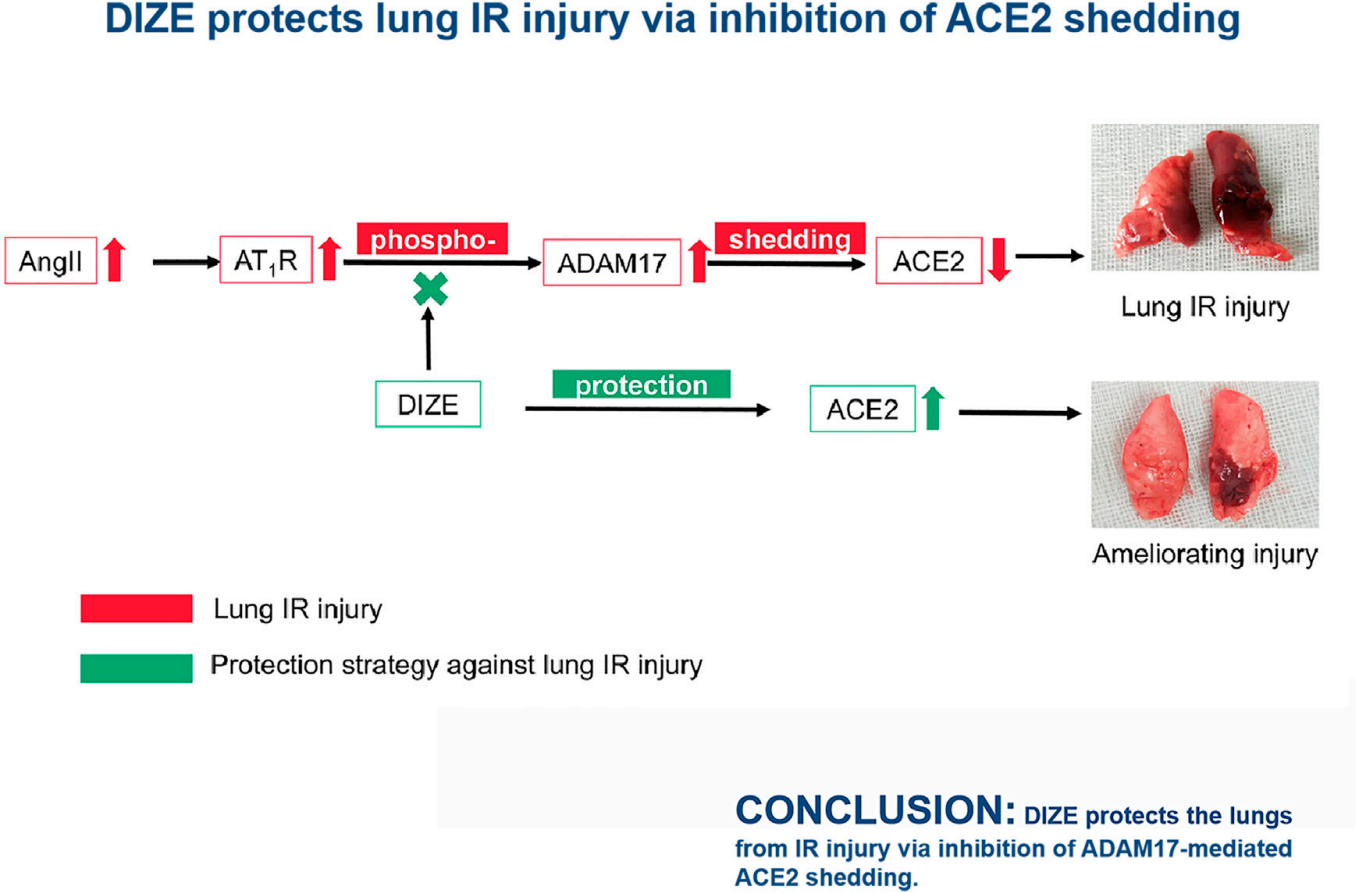


## Introduction

Lung ischemia-reperfusion (IR) injury is the most common cause of postoperative morbidity after lung transplantation (Hsiao et al., 2017), pulmonary thromboendarterectomy (Gormus et al., 2013), and pulmonary hypertension on weaning patients from cardiopulmonary bypass (Ailawadi et al., 2009). Lung IR injury could lead to refractory hypoxemia, pulmonary hypertension, and graft dysfunction after surgery. Once symptoms of lung IR injury develop, mortality could increase threefold (Bates et al., 2015). To date, limited treatments are available for lung IR injury, including protective ventilation strategy (Hsu et al., 2007), prostanoids (Meade et al., 2003), inhaled nitric oxide (Kerr et al., 2012), certain anti-inflammatory approaches (Çakir et al., 2004; Donahoe et al., 2016), and extracorporeal life support (Jin et al., 2013); however, none of these treatments have shown clinical efficacy. The incidence of lung IR injury after lung transplantation is approximately 30% (Lipworth and Dagg, 1994; Taylor et al., 2007). Therefore, treatment of lung IR injury has always been a pressing clinical problem.

**FIGURE 1:**
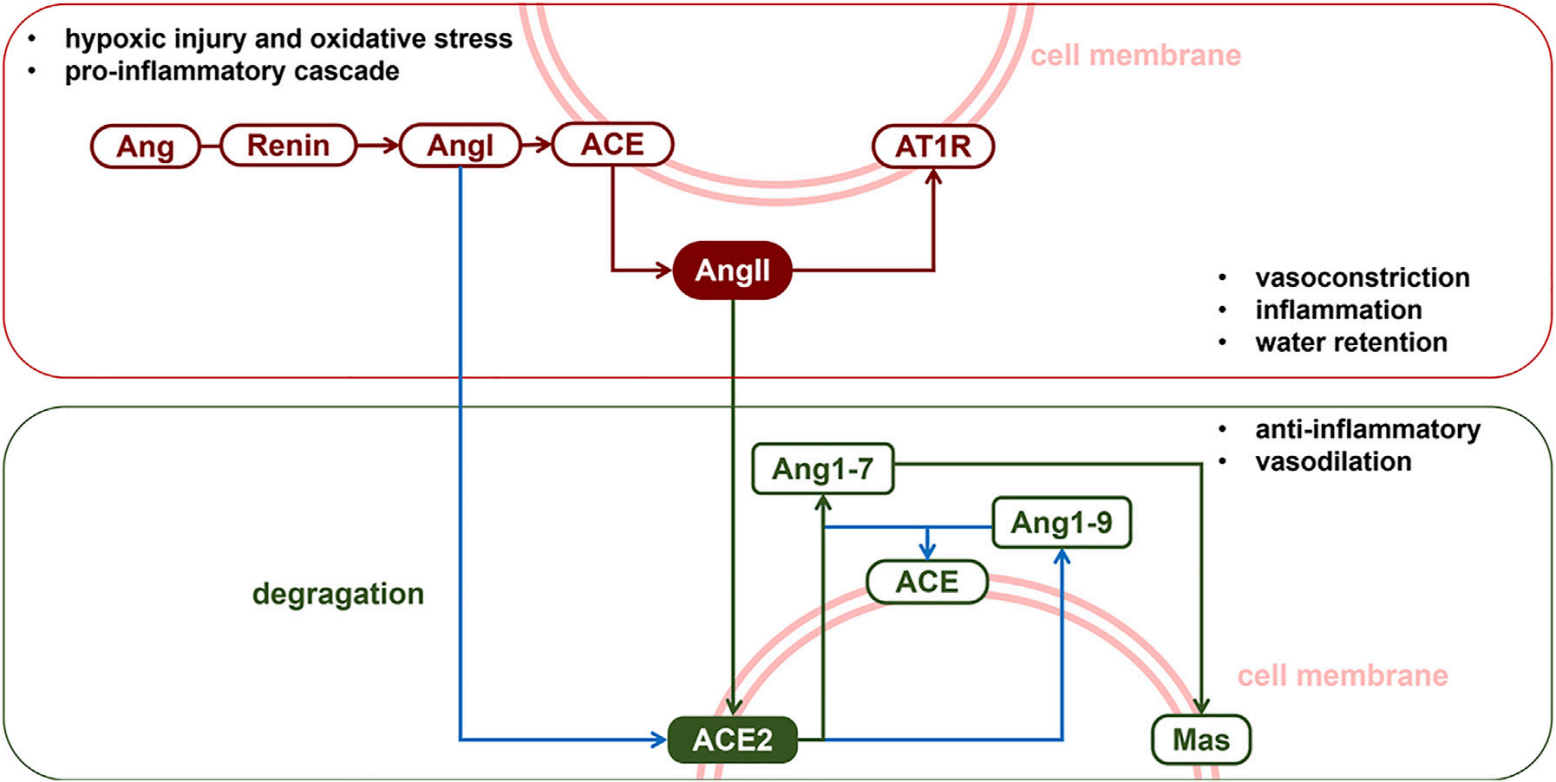
The renin-angiotensin system. The RAS classic axis consists of the membrane protein angiotensin converting enzyme (ACE), vasoconstrictor angiotensin II (AngII) and its specific receptor type1 angiotensin II receptor (AT_1_R). Activation of ACE-AngII-AT_1_R pathway could induce pulmonary vasospasm or aseptic inflammation. Meanwhile, autocrine or paracrine signalling of AngII was counterbalanced by membrane protein ACE2, which, with ACE, degragates AngI and AngII into protein fragments Ang1-7. The ACE2-Ang1-7-Mas pathway, also called the protective arm of RAS, exhibits a protective action in the lungs.

Lung IR injury is characterized by pulmonary vascular constriction, increased microvascular permeability, diffuse alveolar damage, and progressive inflammation within 24–48 h after reperfusion. The pulmonary microvascular endothelial barrier and epithelium constitute the first line of defense against pulmonary IR. Noxious stimulation to the lung is known to result in over-activation of the renin-angiotensin system (RAS), which targets the pulmonary vascular bed (Kehoe et al., 2016). Figure 1 exhibits the classic axis in the RAS system and its physiological function in the lung. Member proteins of RAS are distributed in pulmonary endothelial and epithelial cells. Over-activation of the angiotensin-converting enzyme-angiotensin II-AngII type 1 receptor (ACE-AngII-AT_1_R) pathway is a crucial factor in exacerbating lung injury. Angiotensin II (AngII) is the strongest pulmonary vasoconstrictor in RAS, which is immediately increased following lung reperfusion (Atochina et al., 1997; Cziráki et al., 2002; Balyasnikova et al., 2005; Zhang et al., 2009). There is abundant angiotensin-converting enzyme 2 (ACE2) expressed in the membrane of human pulmonary artery smooth muscle (Jia et al., 2005) and alveolar epithelial cells (Zhao et al., 2010). ACE2 exerts an endogenous pulmonary protective action via the ACE2-Ang1-7-Mas pathway through which it degrades excessive AngII into Ang1-7 (Zhao et al., 2010). In both hypoxia (Peña Silva et al., 2012) and oxidative stress (Patel and Verma, 2020) models, AngII-induced vasoconstriction was exaggerated. It has been reported that endogenous or exogenous ACE2 could counteract AngII and normalize vascular tone. COVID-19 (Prabakaran et al., 2004), SARS-CoV (Liu et al., 2017), and avian influenza A (H_5_N_1_) (Shenoy et al., 2011) all specifically downregulate the expression of ACE2 in alveolar epithelium (Haber et al., 2014) and could induce acute respiratory syndrome via targeted damage of ACE2 in the lung. The literature reviewed above indicated that ACE2 plays an important role in maintaining pulmonary vascular tone and epithelial integrity. Thus, we propose that the function of endogenous ACE2 plays a central role in determining the intensity of lung IR injury.

DIZE is one of the only two existing endogenous ACE2 indirect antagonists. DIZE is an anti-*Trypanosoma cruzi* agent, and its endogenous ACE2 protective function is an off-target treatment effect. The peak time of DIZE is approximately 1–2.5 h, and the eliminated half-life is 100 h (Yao et al., 2002). It has been indicated that single-dose injection of DIZE or continuous medication (from 2 days to 4 weeks) could cause a potential organ protection effect in the heart and kidney under harmful stress (Qi et al., 2013; Shenoy et al., 2013; Velkoska et al., 2016; Li et al., 2018), where the RAS system plays its dominant biological function. Haber et al. (Velkoska et al., 2016) demonstrated that although DIZE could not alter the transcription level of ACE2, it could restrain ACE2 dysfunction and preserve intrinsic ACE2/AngII balance with exposure to the cause of lung injury. Shenoy et al. (2013) reported that intraperitoneal (i.p.) injection of DIZE (15 mg/kg/day) for 2 weeks could downregulate ADAM17 mRNA levels and inhibited the loss of tissue ACE2 in myocardial cells in a chronic heart failure animal model, thus improving heart function by keeping the ACE/ACE2 ratio closer to an optimized profile. Moreover, DIZE was found to improve endothelial dysfunction and vasodilatory function in a pulmonary hypertension model (Li et al., 2018).

In this study, we used both *in vivo* and *in vitro* lung reperfusion models to test the hypothesis that the ACE2 simulator drug DIZE acts as a protective agent in lung IR injury.

## Materials and Methods

### Lung Ischemia-Reperfusion Model in Rats

#### Animals Preparation

All procedures involving animal experimentation were approved by the Animal Research and Care Committee at China-Japan Friendship Hospital Affiliated to Peking University Health Science Center. Thirty-five male Sprague–Dawley rats (250–400 g) were obtained from Beijing Vital River Laboratory Animal, Inc. (Beijing, China). All animals were fed with *ad libitum* access to food and water, treated humanely, and maintained at room temperature (25°C ± 5°C), with a 12 h day-night cycle.

All the rats received an i.p. injection of heparin (50 U/kg) before surgery, and 10 min before the surgery, the animals were anesthetized using an i.p. injection of 10% chloral hydrate (0.3 ml/100 g), with 0.1–0.3 ml additional anesthetic added when necessary. Tracheotomy was performed with the rat in supine position, and the ventilator was set at tidal volume (V_T_) 6–8 ml/kg (<3 ml) and respiratory rate (RR) 80–90 bpm. A thermostatic blanket (Cat. No. HAD-HTP103L; Heng Odd, Beijing, China) was set at 37°C to maintain a constant body temperature. A pulse oxygen saturation (SpO_2_) probe was bound to the right hindlimb to monitor perioperative SpO_2_ and heart rate.

**FIGURE 2:**
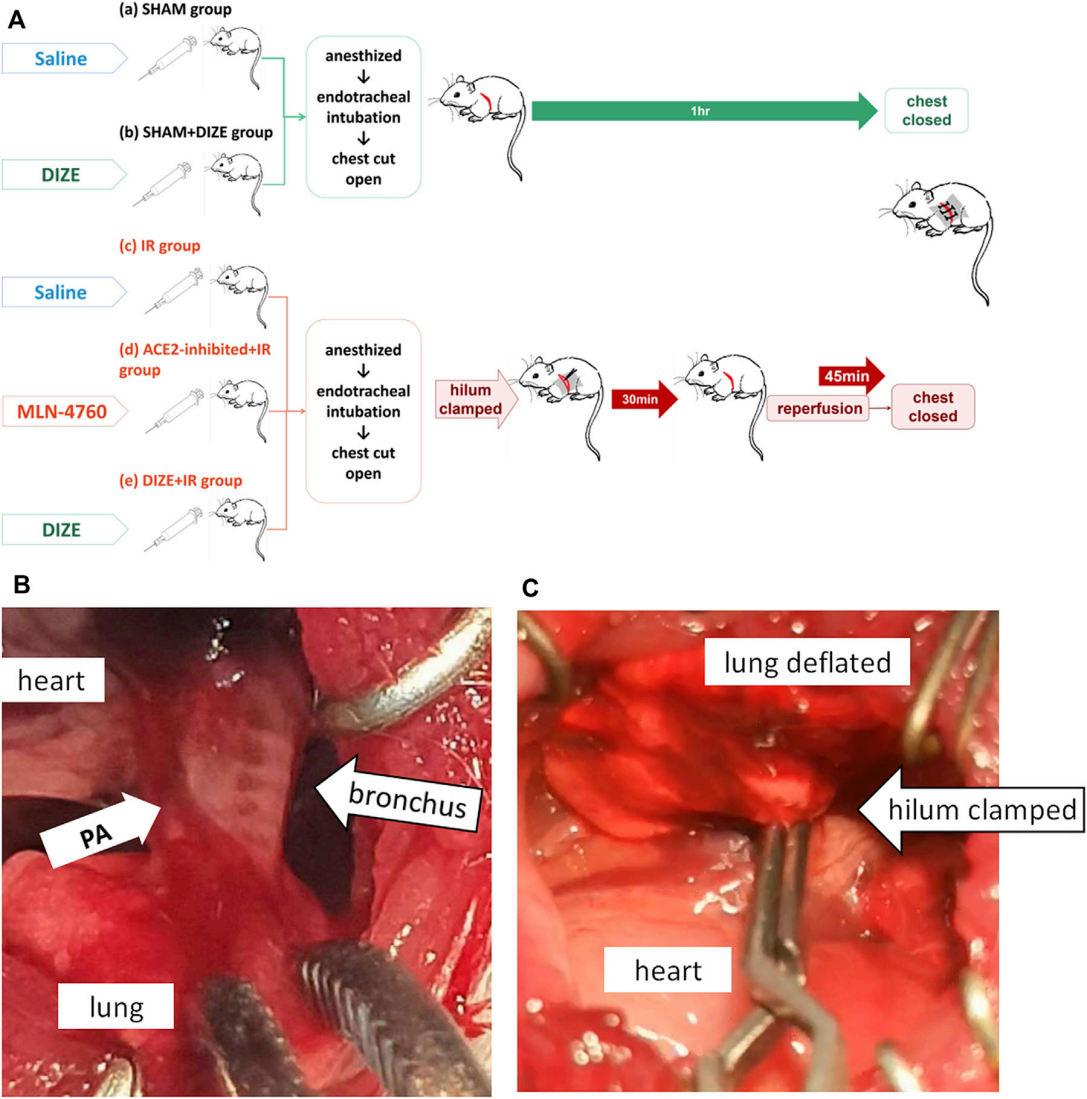
Lung ischemia-reperfusion experimental procedures. **(A)** Animals were randomized into five groups: the *SHAM group*, *SHAM + DIZE group*, *IR group*, *IR + ACE2-inhibited group*, and *IR + DIZE group*. For the *IR + DIZE* and *SHAM + DIZE groups*, 0.1% DIZE 15 mg/kg/day was injected intreperitoneally. For the *IR + ACE2-inhibited group*, a potent ACE2 inhibitor, MLN-4760 was injected via the caudal vein before surgery. For the *IR group*, *IR + ACE2-inhibited group* and *IR + DIZE group*, a lung IR injury model were established. For the *SHAM* and *SHAM + DIZE groups*, only tracheotomy, mechanical ventilation and open chest operation were performed. **(B)** The key procedure during the surgery was to find the bronchus accompanied by the pulmonary artery. **(C)** The lung deflated when the hilum was clamped.

Animal grouping and intervention techniques are shown in Figure 2A. Animals were randomized into five groups: *SHAM, SHAM + DIZE, IR, IR + ACE2-inhibited,* and *IR + DIZE group*, with *n* = 7 for each group. For the *SHAM group*, 0.9% saline (1 ml/kg) was infused i.p. for 7 days before surgery. Animals in the *SHAM group* received both general anesthesia and tracheotomy, and then their chests were opened for an hour, before being closed up. For the *SHAM + DIZE group*, ACE2 protectant 0.1% DIZE (Cat. No. 536-71-0; Sigma-Aldrich, Australia; 15 mg/kg) was injected i.p. once a day for a week (Qi et al., 2013; Li et al., 2017). Then, the animals each received general anesthesia and thoracic surgery. For the *IR group*, 0.9% saline (1 ml/day) was infused i.p. for 7 days before surgery. After anesthesia, the chest was opened, and the left hilum was isolated. We used a microvascular clip to clamp the hilum for 30 min, followed by 45 min of reperfusion with the chest closed (detailed process described in the next paragraph). For the *IR + ACE2-inhibited group*, ACE2 inhibitor compound MLN-4760 (Cat. No. 305335-31-3; Merck, Millipore, United States) was dissolved in 10% DMSO and injected (1 mg/kg) (Soler et al., 2007) once a day for 2 days via the tail vein, with the last dose given within 30 min before surgery (Ye et al., 2012; Li et al., 2016; Hashimoto et al., 2019). The animals’ left hila were then occluded and reperfusion was performed. For the *IR + DIZE group*, after i.p. of 0.1% DIZE (15 mg/kg) once a day for a week, the animals’ left hila were occluded and reperfusion was performed.

#### Lung IR Injury Model in Rats

For the lung IR injury model, a surgical incision was made at the point where heart movement was the most pronounced. The muscle was then carefully separated layer by layer and the pleura entered. The heart was gently pushed toward the right, and the lung was retracted laterally to expose the left main bronchus. The key surgical techniques in lung IR injury model are shown in Figures 2B,C. The bronchial rings were found to act as the anatomical landmark of the left hilum (Figure 2B), and the ventilator was paused if necessary. A noninvasive microvascular clip was used to clamp the hilum, ensuring that the left lung deflated during ventilation (Figure 2C). The clamp was removed after 30 min of ischemic phase, followed by 45 min of reperfusion and double-lung ventilation, and then the chest was closed.

After 45 min of reperfusion, blood was extracted from the abdominal aorta for arterial blood gas (ABG) analysis. All animals were euthanized by exsanguination. The middle lobe of the right lung was harvested and stored below −80°C for Western blot analysis. The posterior lobe of the right lung was stored in 10% formalin for hematoxylin and eosin (H&E) staining. The anterior lobe and the accessory lobe of the right lung were harvested for wet/dry weight ratio (W/D) measurement. The left lung was stored in 4% paraformaldehyde for 24 h for immunohistochemistry (IHC) analysis.

### Cardiac Catheterization and Monitoring

A rodent pressure catheter (Transonic Science, Hong Kong, China) was used for cardiac catheterization. A 24-G intravenous trocar was inserted into the carotid artery to monitor the heart rate and arterial blood pressure. A 22-G intravenous trocar was inserted into the internal jugular vein. The pressure catheter (1.9 F) was inserted into the trocar and advanced into the right ventricle and pulmonary artery to monitor the central venous pressure and pulmonary hemodynamics.

### Wet/Dry Weight Ratio Measurement

The wet/dry weight ratio (W/D) measurement was used for the evaluation of the severity of intra-alveolar edema in the rat lung (Fehrenbach et al., 2014). The samples were rinsed with normal saline, dried with filter paper, and then weighted for wet weight. The tissue was dried at 70°C for 12 h and weighed for dry weight. The ratio of wet/dry (W/D) weight was calculated to determine the extent of pulmonary edema.

### Histology and Pathology

After gross specimen observation, the lungs were embedded in paraffin wax, cut into thin slices, and stained with H&E. The Lung Injury Score (LIS) (Table 1) could reflect the degree of lung tissue injury under the condition of lung transplantation or lung IR injury in rat lung (Pirat et al., 2006; Hashimoto et al., 2019). For each sample, five randomized fields were chosen at high magnification (×400). A pathologist blinded to the animal grouping was responsible for estimation of the LIS. After the overall observation of the five randomized fields, the researcher quantified the extent of the lesion according to each item (neutrophil infiltration or aggregation, airway epithelial cell damage, interstitial edema, hyaline membrane formation, and hemorrhage). Scores were quantified as follows, for each item: normal (score = 0), the area of lesion <20% field of vision; minimal (score = 1), the area of lesion 20–30% field of vision; mild (score = 2), the area of lesion 30–50% field of vision; moderate (score = 3), the area of lesion 50–80% field of vision; and severe (score = 4), the area of lesion 80–100% field of vision. The total value of LIS was recorded for quantitative data for the assessment of lung histological injury.

**TABLE 1:** Lung injury score (LIS) Pirat et al., 2006.

		Normal	Minimal	Mild	Moderate	Severe
1	neutrophil infiltration or aggregation					
2	airway epithelial cell damage					
3	interstitial edema					
4	hyaline membrane formation					
5	hemorrhage					

Score (each item): normal = 0; minimal = 1; mild = 2; moderate = 3; severe = 4.

Scoring: normal (score = 0): the area of lesion <20% field of vision; minimal (score = 1): the area of lesion 20–30% field of vision; mild (score = 2): the area of lesion 30–50% field of vision; moderate (score = 3): the area of lesion 50-80% field of vision; severe (score = 4): the area of lesion 80–100% field of vision.

### Immunohistochemical Analysis

Lung tissue was fixed in 4% paraformaldehyde. 3,3-Diaminobenzidine (DAB) staining for AngII and ACE2 was performed on paraffin section of tissue samples. Treatment of the tissue section followed deparaffinization in xylene (15 and 15 min) and rehydration through 100, 85, and 75% ethanol (each phase for 5 min) were performed. Antigens were retrieved by Tris-EDTA (pH 8.0, Cat. No. G1206-250ML, Servicebio, China) in a microwave (medium heat, 8 min; cooling, 8 min; low heat, 7 min). After natural cooling, slices were washed in PBS. Endogenous peroxidase activity were blocked by 3% hydrogen peroxide at room temperature (for 25 min), avoiding light, then the samples were washed in PBS. 3%BSA was added to the sections and sealed for 30 min at room temperature. After removing the serum sealing solution, primary antibodies for ACE2 (1:1,000, Cat. No. sc-390851, Santa Cruz, California, USA) and AngII (1:1,000, Cat. No. yb005Mu01; YBscience, Shanghai, China) were added to the samples respectively, and were incubated at 4°C overnight. After washed in PBS, 50 μl HRP-labled secondary antibody (1:200, Cat. No. G1213, Servicebio, Wuhan, China) were added to the section, then incubated at room temperature for 50 min. After washed in PBS, 50 μl DAB color developing reagent (1:50, Cat. No. G1215, Servicebio, Wuhan, China) was added on the samples. Nucleus staining was colored with hematoxylin for several seconds. Sections were dehydrated and mounted following 75, 85, 100, 100% ethanol, n-butanol and xylene (each phase for 5 min) until they were transparent. Sections were dried and mounted with neutral gum.

The result of IHC staining was analyzed using Image Pro-Plus 6.0 software. The whole tissue section was scanned under the light microscope, and then five randomized fields were chosen at ×400magnification. The brown-stained area around blue-stained cell nuclear was defined regions of interest (ROI). After delineating ROI, integrated optical density (IOD) and area were measured in each field. The mean optical density (IOD/area) represented the concentration of the specific protein in the area of interest.

### Western Blot Analysis

The protein samples (25 μg) were separated on 10% SDS-PAGE gels and were transferred onto PVDF membrane. The membranes were then blocked with 5% non-fat milk solution in TBST at room temperature for 2 h and then incubated with primary rabbit anti-rat antibody against AT_1_R (1:400; Cat. No. SAB3500209, Sigma-Aldrich, Darmstadt, Germany), a disintegrin and metalloprotease 17 (ADAM17) (1:1,000; Cat. No. ab 2051; Abcam, Cambridge, UK), p38MAPK (1:800; Cat. No. ab47363; Abcam), p65NF-κB (1:500; Cat. No. ab97726; Abcam), IL-1β (1:800; Cat. No. ab9722, Abcam), IL-6 (1:1,000; Cat. No. ab9324, Abcam), TNF-α (1:1,000; Cat. No. sc-52746, Santa Cruz), or β-actin (1:1,000; Cat. No. ab8226; Abcam) overnight at 4°C. After three washes with TBST, samples were incubated with HRP-conjugated anti-rabbit IgG at room temperature for 1 h. After having washed with TBST, blots were detected using ECL kit (Tiangen Biotech, China), and the target band was visualized using the ChemiDoc imaging system (Bio-Rad, United States). Quantity One software (Bio-Rad, United States) was used to detect protein levels. Relative expression of the protein was quantified by IOD value of the protein band (normalized to β-actin). The tests were repeated three times independently in each group.

### Oxygen-Glucose Deprivation/Reperfusion Model *in vitro*

A549 pulmonary epithelial cells were provided by the Institute of Basic Medicine, (Chinese Academy of Medical Sciences, Beijing, China) and normally cultured in Dulbecco’s Modified Eagle Medium (DMEM) (Cat. No. 11966025, Gibco, Shanghai, China) containing 10% fetal bovine serum (FBS). Cells were plated at a density of 5×10^4^ cells/mL and incubated in humidified atmosphere at 37°C with 5% CO_2_. After 24 h of incubation, for the *Control group*, the cells were incubated in standard culture for 8 h. For the *OGD/R groups*, cells were placed in glucose-free medium and incubated in 1% O_2_:5% CO_2_:94 %N_2_ for 8 h. OGD was terminated by replacing the exposure medium with normal feeding medium incubated under normal condition at 37°C with 5% CO_2_:95% room air of reoxygenation for 12 h. For the *treatment groups*, A549 were pre-incubated with different concentrations of DIZE (0.01, 0.1, 0.5 μM) for 24 h. For the *ACE2 inhibition groups*, A549 were pre-incubated with different concentrations of ACE2 inhibitor DX600 (0.5, 1, 5 μM) (Cat. No. AS-62337; AnaSpec, Fremont, United States) for 24 h (Ye et al., 2012).

### Cell Viability

The effect of OGD/R on A549 survival and the intervention effect of DIZE or DX600 (ACE2 inhibitor) on OGD/R cells were measured using cell counting kit-8 (CCK-8, Dojindo Biochem, Shanghai, China). Cells of different groups were seeded in 96-well plates with 5×10^4^ cells/ml and incubated for 24 h; then 10 μl of CCK-8 solution was added to each well, and the cells were incubated for 4 h. The optical density (OD) of each well was measured at 450 nm. Cell viability was calculated as (OD_test_-OD_base_)/(OD_control_-OD_base_) × 100%, where OD_base_ was the OD of wells without cells.

### Detection of Intracellular Reactive Oxygen Species

Intracellular production of ROS was tested using the Reactive Oxygen Species Assay Kit (Cat. No. KGT010-1, Keygentec, Nanjing, China). Fluorescent probe 2′7′-dichlorofluorescein acetate (DCFH-DA) was applied for ROS detection. Intracellular ROS could oxidize non-fluorescent DCFH, thus producing fluorescent DCF, which could be detected. Dilution of DCFH-DA with serum-free cell medium at a concentration of 10 μM was performed. Cells were covered with DCFH-DA and incubated for 20 min. Serum-free medium was used to wash the cells three times and remove extracellular DCFH-DA. The fluorescence microplate reader (Molecular devices, United States) was used to measure DCF density at Ex/Em 488/525 nm, and H_2_O_2_ (100 μM) was added to cells to get the maximum DCF density. The level of ROS in each group of cells was calculated as a percentage of maximum DCF density.

### Detection of Intracellular Reactive Nitrogen Species

Intracellular production of RNS was detected via the RNS detection kit (Cat. No. HR8791, BaiAoLaiBo, Beijing, China). The fluorescent probe O52 could penetrate the cell membrane and would be hydrolyzed as O52D. The reaction of O52D and RNS produced strong fluorescence O52F at Ex/Em 490/516 nm.

O52 was mixed with serum-free cell medium in 1:1,000 dilution at 37°C. The culture medium was removed from the cells and the working solution containing O52 probe was added to the cells. After incubating for 30 min, the cells were washed three times. The fluorescence intensity at 516 nm was correlated with the concentration of intracellular RNS.

### Detection of ACE2 Enzyme Activity

ACE2 enzyme activity was detected via an Angiotensin II-Converting Enzyme (ACE2) Activity Assay Kit (Cat. No. K897-100, Biovision, United States). The enzyme activity of ACE2 was quantified as the cleavage rate of the substrate: free fluorescent fragment 4-amino-methoxycoumarin (MCA). We prepared 0, 50, 100, 150, 200, and 250 pmol/well of MCA-Standard mixed with ACE2 Assay Buffer. The fluorescence was measured at Ex/Em 320/420 nm, and then the MCA-Standard curve was obtained, and the slope of the curve (△RFU/pmol) was calculated. One million A549 cells were blended with 400 μl ACE2 Lysis Buffer for 10 min at 4°C and centrifuged at 16,000 RPM for 10 min, after which the supernatant was collected. A BCA Protein Assay Kit (Cat. No. KGPBCA, KeyGEN BioTECH, Nanjing, China) was applied for protein quantification. We added samples in the wells ordered as 2 μl of ACE2 Lysis buffer in the plate for Background Control (BC); 2 μl of Positive Control (PC); 2 μl of cell lysate sample to be tested; and 2 μl of Negative Control (NC). The wells were filled with ACE2 Assay Buffer for a total volume of 50 μl at room temperature. From 30 min to 2 h post-incubation, the fluorescence was dynamically measured at Ex/Em 320/420 nm. The amount of released MCA was obtained based on the standard curve MCA=(RFU1-RFU2)/(t1-t2). ACE2 enzyme activity was calculated using the following formula: ACE2 activity = △MCA/(t1-t2)/Sample protein (mg).

### Statistical Analysis

IBM SPSS Statistics (Version 23.0; Manufacturer, IBM Corp., NY, United States) was used for statistical analysis. Quantitative results were presented as mean ± SEM. One-way analysis of variance (ANOVA) was applied for comparison between groups. If equal variances are assumed, Tukey’s test was used for post-hoc pairwise comparison. If equal variances are not assumed, Dunnett’s test was used for pairwise comparison. *p <* 0.05 was considered statistically significant.

## Results

### Perioperative Monitoring Testified to the Establishment of Lung IR Injury

**FIGURE 3:**
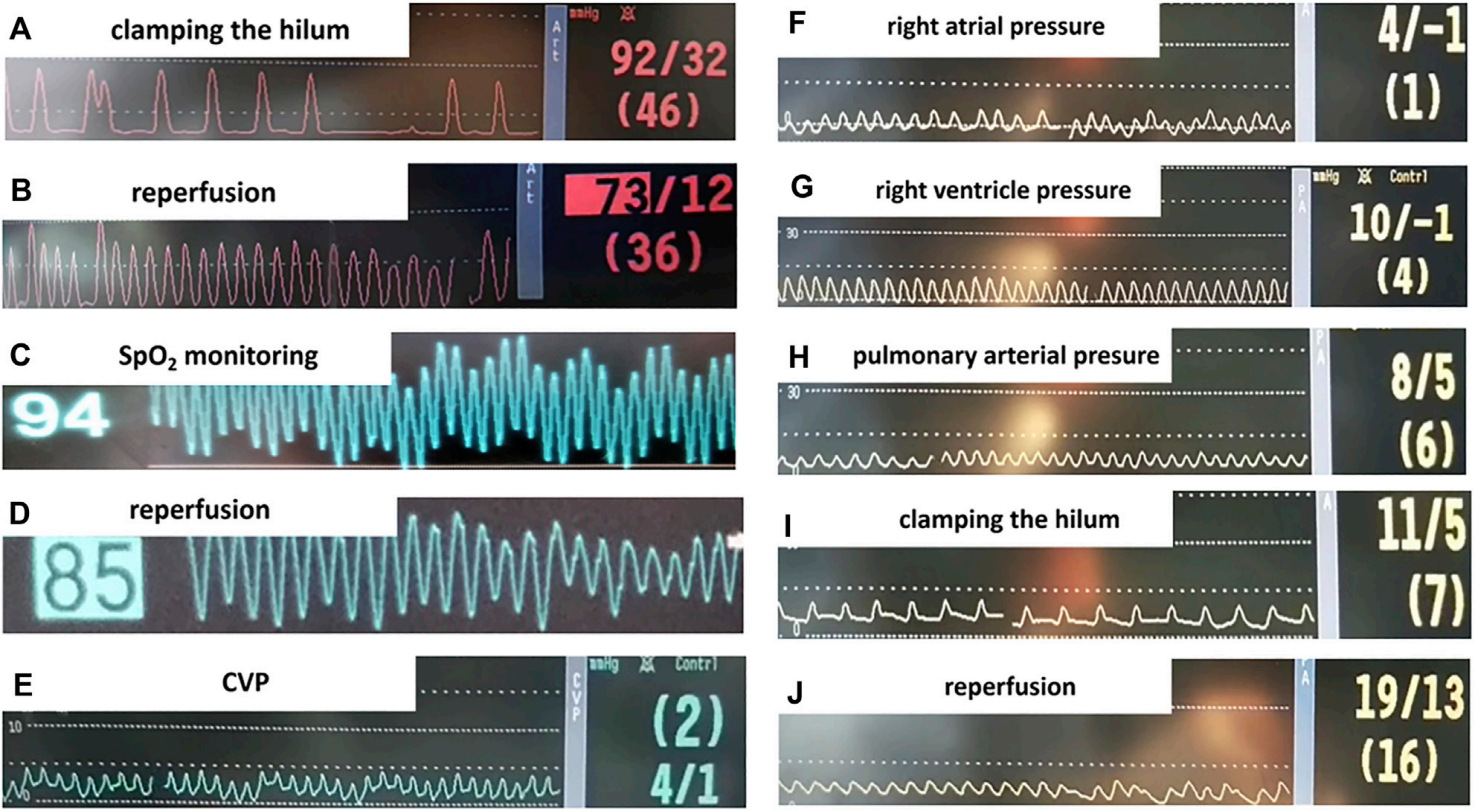
Monitoring parameters during surgery. **(A,B)** Systemic blood pressure before and after we clamped the hilum. **(C,D)** SpO_2_ before and after lung reperfusion. **(E-J)** Right heart catheterization (RHC) in the rat lung IR model and intraoperative monitoring. **(E)** Central venous pressure (CVP) measured by RHC. **(F)** Right atrial pressure. **(G)** Right ventricle pressure. **(H**) Pulmonary arterial pressure (PAP). **(I)** PAP when left lung hilum clamped, the animal presented bradycardia with PAP slightly elevated. **(J)** Elevated PAP during lung reperfusion.

Cardiac catheterization and monitoring helped to guarantee the safety and quality of the animal model. As shown in Figures 3A,B and Figures 3H–J, bradycardia was common when the pulmonary artery was occluded. Consistent with clinical manifestation, characteristic changes in hemodynamics included desaturation (Figures 3C,D), hypotension (Figures 3A,B), and an increase in pulmonary arterial pressure (Figures 3H–J) were seen during the reperfusion phase.

### DIZE Ameliorated Lung Edema and Pathological Changes, Improved Oxygenation Index During Lung IR Injury

**FIGURE 4:**
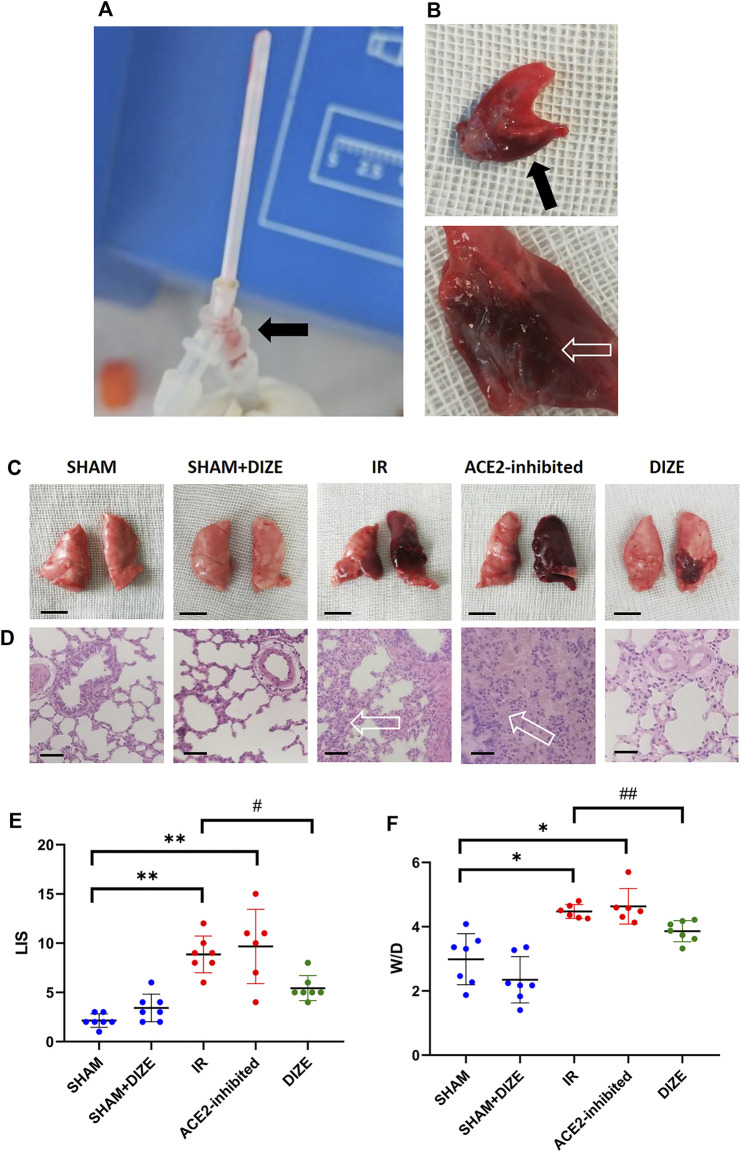
Manifestation of lung ischemia-reperfusion (IR) injury. **(A)** The experimental illustration. The experiment animal produced pink frothing phlegm (arrow) in the endotracheal tube. **(B)** The cut surface of the lung revealed acute pulmonary edema and congestion (arrow) in the IR rat model. **(C)** Gross view in different groups (scale bar 1 cm). **(D)** HE staining (×400 magnification); arrow-massive inflammatory cells and pink liquid secretion in the alveolar space and alveolar septum, resulted in severe destruction of the alveolar structure (scale bar 50 µm). **(E)** Lung injury score (LIS) in different groups. **(F)** Wet/Dry ratio. Data were expressed as mean ± SEM. *p < 0.05, **p < 0.01 vs. SHAM group; #*p* < 0.05, ##*p* < 0.01 vs. IR group, *n* = 7.

As shown in Figures 4B–D, gross and histopathological examination revealed that lungs in the *SHAM* and *SHAM + DIZE groups* showed the appearance of normal lung tissue. Lung tissues in the *IR group* turned dark red, indicating congestion and edema. Pathological findings in the *IR group* included inflammatory cell infiltration in pulmonary interstitium and alveolar space and destruction of alveolar structure. Several rats in the *IR* and *IR + ACE2-inhibited group* produced pink frothy sputum in the endotracheal tube (Figure 4A), consistent with previous research (Salem et al., 2014) and clinical manifestation of acute pulmonary edema. In the *IR + ACE2-inhibited group*, the whole left lung showed severe congestion and consolidation. HE staining demonstrated the disappearance of normal lung structure, replaced by massive inflammatory cell infiltration and pink liquid secretion in the alveolar space, indicating severe, diffuse alveolar damage. In the *IR + DIZE group*, lungs demonstrated mild to moderate alveolitis, where normal lung tissue existed among inflammatory cell infiltration foci and mildly thickened alveoli septum.

As shown in Figure 4E, the *SHAM* and *SHAM + DIZE groups* exhibited mild lung injury, whereas the IR groups reached moderate to severe degrees of lung injury (LIS score *SHAM* vs. *IR group*, *p <* 0.01). Inhibition of ACE2 via MLN-4760 achieved moderate to severe lung injury (LIS score *SHAM* vs *IR + ACE2-inhibited group*, *p <* 0.01), though there was no statistically significant difference when compared to *IR group*. DIZE could significantly reduce the degree of lung injury pathological scoring during lung IR injury (LIS score *IR* vs. *IR + DIZE group*, *p <* 0.05). The W/D ratio is shown in Figure 4F. The W/D ratio in the *IR group* (W/D = 4.48 ± 0.22) and *IR + ACE2-inhibited group* (W/D = 4.63 ± 0.55) was significantly higher than in the *SHAM group* (W/D = 2.99 ± 0.80; W/D *SHAM* vs. *IR group, SHAM* vs. *IR + ACE2-inhibited group*, *p <* 0.05, respectively), whereas DIZE could significantly alleviate pulmonary edema after IR injury (*IR + DIZE group* W/D *=* 3.86 ± 0.33, *p <* 0.05, vs. *IR group*).

Analysis of ABG is shown in Table 2. Compared to the *SHAM group*, a significant decrease in PaO_2_, SaO_2_, and oxygenation index (OI) was noted in the *IR group* (PaO_2_ *SHAM* vs. *IR group*, *p <* 0.01; SaO_2_ *SHAM* vs. *IR group*, *p <* 0.05; OI *SHAM* vs. *IR group*, *p <* 0.01). There was a certain trend; however, there was no statistical difference indicating that inhibition of ACE2 might deteriorate the decrease of PaO_2_ and OI during IR injury (PaO_2_ *IR* vs. *IR + ACE2-inhibited group*, 69.8 ± 12.6 mmHg vs. 50.1 ± 13.0 mmHg, *p = 0.075*; OI *IR* vs. *IR + ACE2-inhibited group*, 332.5 ± 59.9 mmHg vs. 238.6 ± 61.8 mmHg, *p = 0.078*). Evaluating from the most important physiological indicator of respiratory function, during lung IR injury, DIZE could significantly increase PaO_2_ and OI (PaO_2_ *IR + DIZE* vs. *IR group*, 96.4 ± 14.0 mmHg vs. 69.8 ± 12.6 mmHg, *p <* 0.01; OI *IR + DIZE* vs. *IR group*, 459.2 ± 66.4 mmHg vs. 332.5 ± 59.9 mmHg, *p <* 0.01), and a possible increase of SaO_2_, but without statistical significance (SaO_2_ *IR + DIZE* vs. *IR group*, 90.8 ± 6.4 vs. 75.9 ± 9.8, *p =* 0.058).

**TABLE 2:** Parameters of arterial blood gas in different groups.

	SHAM	SHAM + DIZE	IR	IR + ACE2 inhibited	IR + DIZE
pH	7.39 (0.09)	7.21 (0.10)	7.10 (0.22)^*^	7.32 (0.13)	7.26 (0.11)
PaCO_2_(mmHg)	35.7 (10.6)	36.7 (4.2)	45.4 (33.7)	42.9 (12.8)	38.8 (10.3)
PaO_2_(mmHg)	110.4 (12.2)	116.7 (13.4)	69.8 (12.6)^**^	50.1 (13.0)^**^	96.4 (14.0)^##^
SaO_2_ (%)	93.1 (3.0)	95.7 (4.0)	75.9 (9.8)^*^	65.9 (16.4)	90.8 (6.4)
OI(mmHg)	545.4 (59.9)	555.8 (63.7)	332.5 (59.9)^**^	238.6 (61.8)^**^	459.2 (66.4)^##^

OI, oxygenation index. OI(mmHg) = PaO_2_/FiO_2._ FiO_2_ = 0.21. Data are expressed as mean ± SEM.

SaO_2_: *IR + DIZE group* vs. *IR group*, 90.8 ± 6.4 vs. 75.9 ± 9.8, *p* = 0.058.

^*^ *p<* 0.05.

^**^ *p<* 0.01 vs. *SHAM group*.

^#^ *p<* 0.05.

^#^ *p<* 0.01 vs. *IR + DIZE group*, *n* = 7.

### DIZE Preserved Endogenous ACE2 in Lung Tissue During Lung IR Injury

**FIGURE 5:**
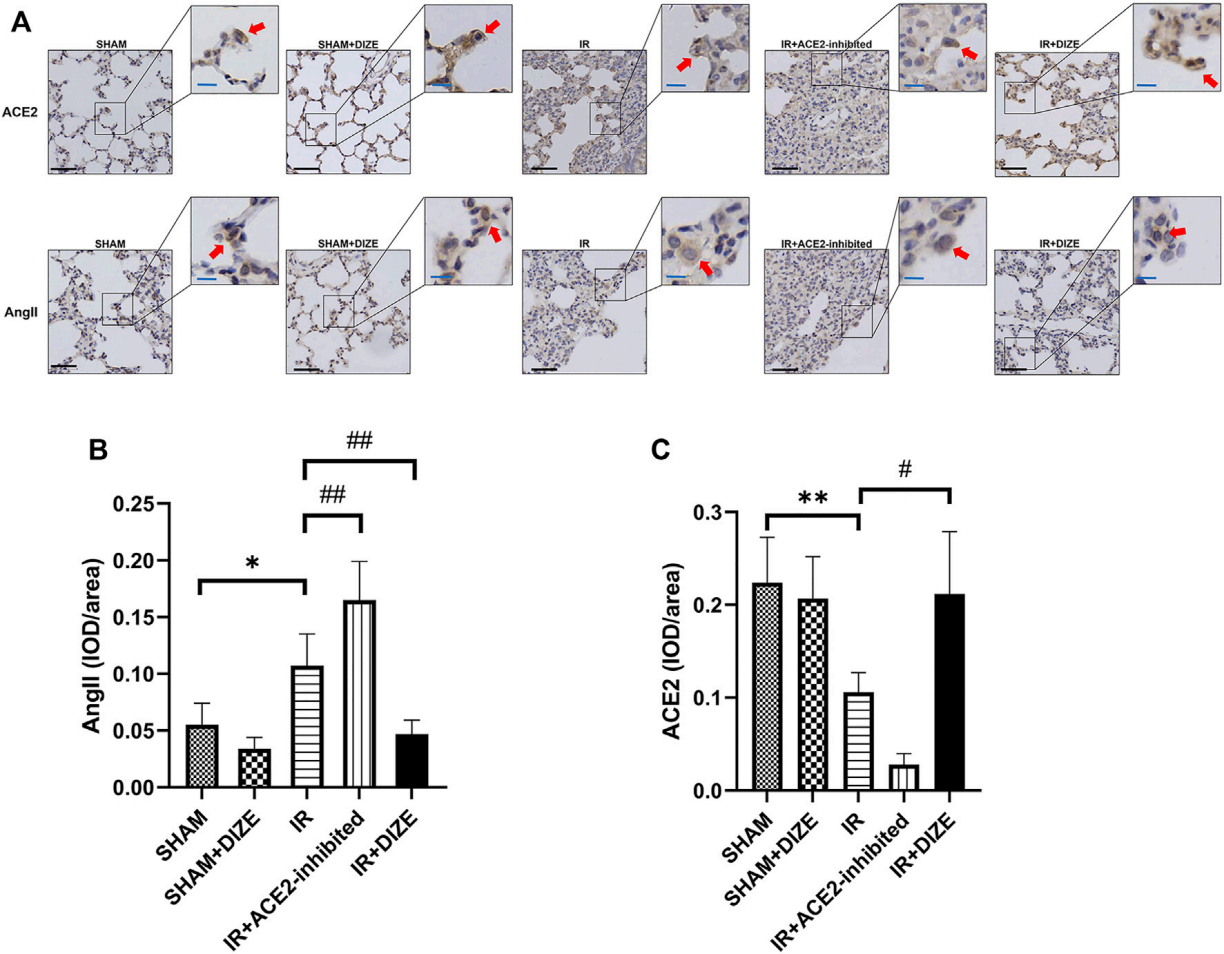
Expression of ACE2 and AngII detected by immumohistochemical (IHC) staining in rat lungs. DIZE significantly decreases AngII expression in lung tissue, and preserved a higher level of endogenous ACE2 expression during IR injury. **(A)** IHC staining results of ACE2 and AngII in rat lung (lower left, black scale bar 50 μm, upper right, blue scale bar 20 μm). **(B)** Semi-quantification of AngII expression (post-hoc analysis: Dunnett’s T3 test). **(C)** Semi-quantification of ACE2 expression (post-hoc analysis: Dunnett’s T3 test). Mean density = IOD/area, which represented the relative concentration of the protein in the area of interest. Data were expressed as mean ± SEM (*n* = 7; representative images are shown). *p < 0.05, **p < 0.01, ***p < 0.001 vs. *SHAM group*; ^*#*^ *p <* 0.05, ^*##*^ *p <* 0.01, ^#*##*^ *p <* 0.001 vs. *IR group*.

The expression and localization of ACE2 and AngII in lung tissue were determined by immumohistochemical (IHC) staining. As shown in Figure 5A, expression of ACE2 and AngII in the lung tissue was located in the alveolar epithelial cells and pulmonary vessel wall. IHC examination revealed the perinuclear expression of AngII and ACE2 (brown). AngII mainly exhibited cytoplasmic immunoreactivity in rat lung, while ACE2 exhibited membranous immunoreactivity.

A semi-quantitative comparison of protein expression demonstrated a significant increase of AngII expression and a significant decrease of ACE2 during IR injury (Figures 5B,C) (mean density of AngII, *SHAM* vs. *IR group*, *p < 0.05*; mean density of ACE2, *SHAM* vs. *IR group*, *p < 0.01*). DIZE could not increase the expression level of ACE2 under normal physiological conditions (mean density of ACE2, *SHAM* vs. *SHAM + DIZE group, p > 0.05*). However, DIZE treatment showed a relatively higher level of ACE2 expression under the circumstances of IR injury (mean density of ACE2 *IR* vs. *IR + DIZE group*, *p <* 0.05), as well as decreased AngII expression levels during IR injury (mean density of AngII, *IR* vs. *IR + DIZE group*, *p <* 0.01).

### DIZE Alleviated ADAM17 Associated AngII-AT_1_R/ACE2 Unbalance in Lung IR Injury

**FIGURE 6:**
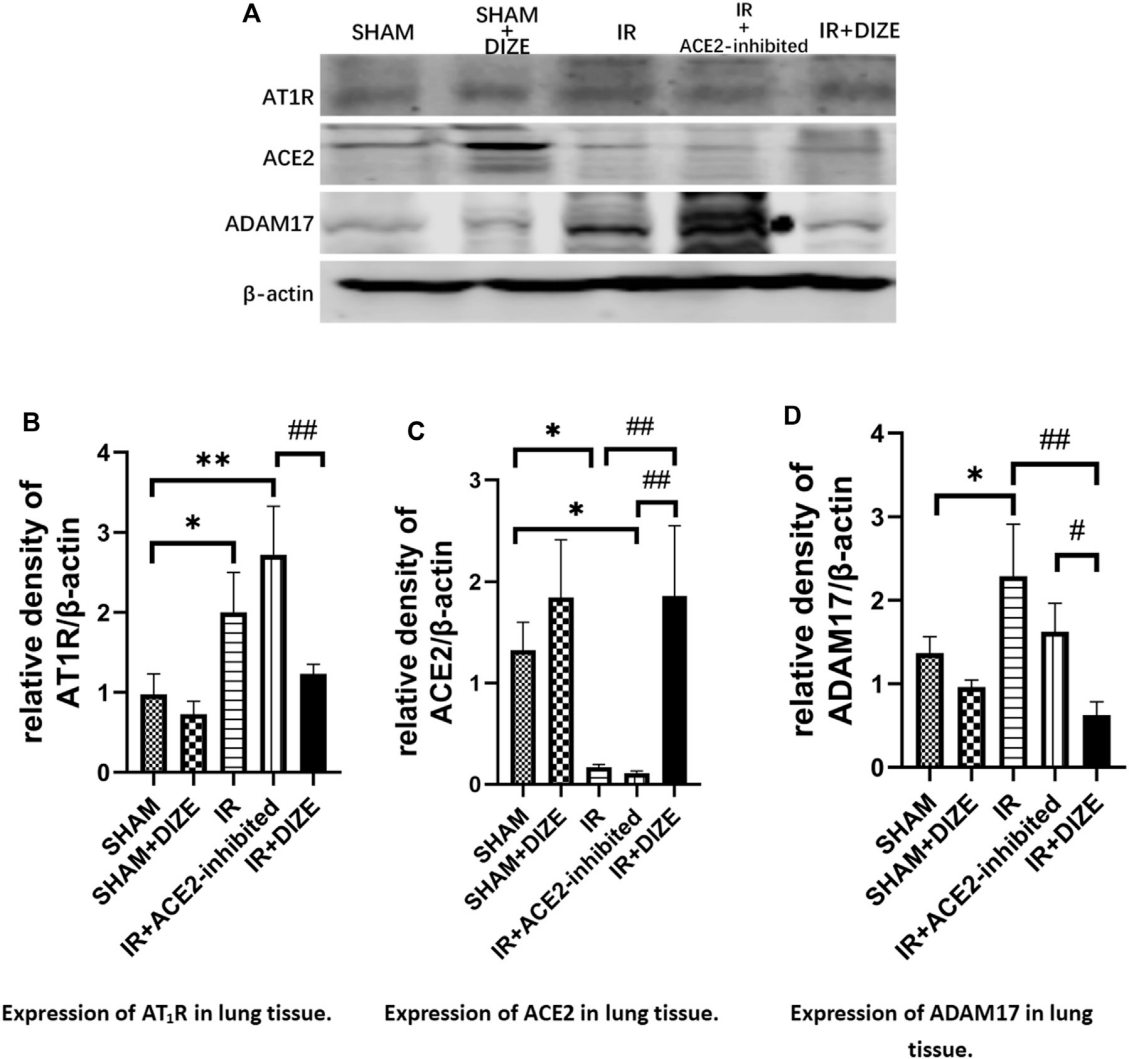
DIZE reduced ADAM17 expression after IR injury and protected local ACE2 during lung IR injury. **(A)** Western blot analysis of AT_1_R, ACE2 and ADAM17 expression. **(B)** Relative quantitative expression of AT_1_R in rat lung (post-hoc analysis: Tukey’s test). **(C)** Relative quantitative expression of ACE2 in rat lung (post-hoc analysis: Tukey’s test). **(D)** Relative quantitative expression of ADAM17 in rat lung (post-hoc analysis: Tukey’s test). Data were expressed as mean ± SEM(*n* = 7), *p < 0.05, **p < 0.01, ***p < 0.001 vs. *SHAM group*; ^*#*^ *p <* 0.05, ^*##*^ *p <* 0.01, ^#*##*^ *p <* 0.001 vs. *IR group*.

The relative protein level of AT_1_R, ACE2, and ADAM17 in lung tissue was examined using Western blot analysis. As shown in Figure 6, consistent with immunofluorescence results, the expression of local ACE2 in lung tissue notably decreased during IR injury (level of ACE2 expression *IR* vs. *SHAM group*, *p < 0.05*), whereas AT_1_R relatively increased during IR injury (*IR* vs. *SHAM group*, *p* < 0.05). DIZE pretreatment preserved higher ACE2 protein levels under the circumstance of IR injury (level of ACE2 expression *IR + DIZE* vs. *DIZE group*, *p < 0.01*).

The expression of ADAM17 in the *IR group* was significantly higher than the *SHAM group* (*p <* 0.05). However, DIZE reduced ADAM17 expression compared to the *IR group* (level of ADAM17, *DIZE* vs. *IR + DIZE group*, *p <* 0.01), thus preserving the protective effect of ACE2 during IR injury.

### DIZE Ameliorated Inflammatory Process in Lung IR Injury

**FIGURE 7:**
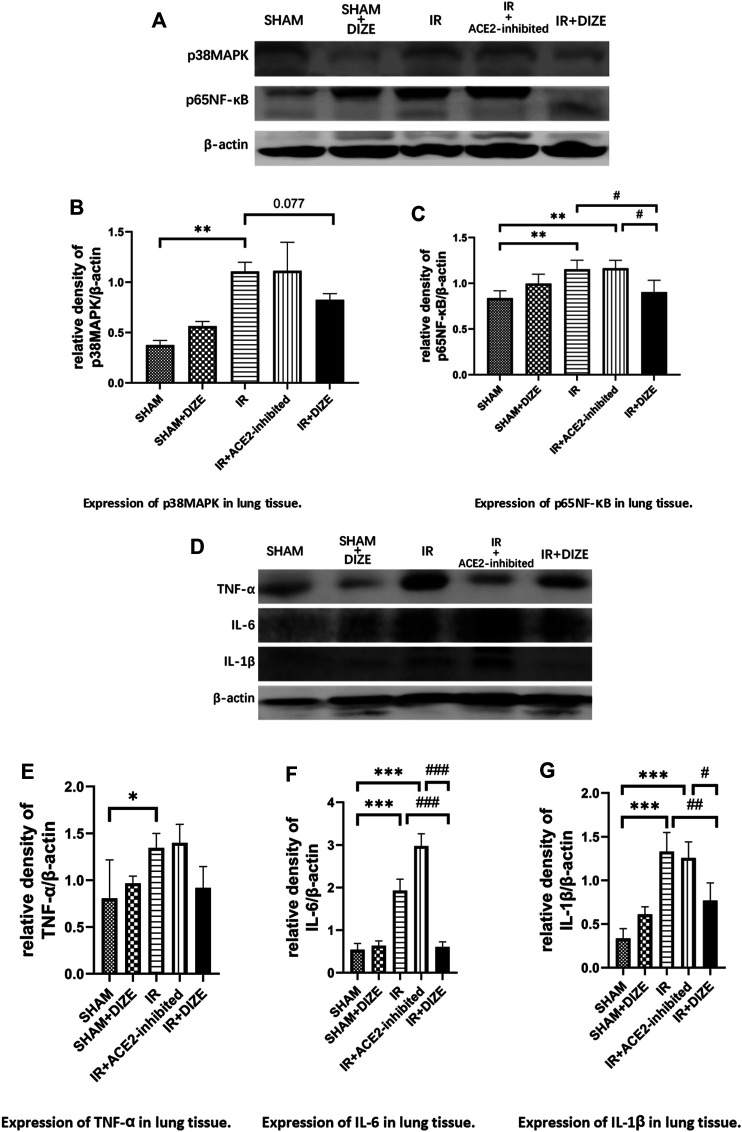
DIZE could significantly lower inflammatory markers IL-6 and IL-1β during the early stage of lung IR injury. **(A)** Western blot analysis of p38MAPK-NF-κB signaling pathway in rat lung. **(B)** Expression of p38MAPK in lung tissue (post-hoc analysis: Dunnett’s T3 test). **(C)** Expression of p65NF-κB in lung tissue (post-hoc analysis: Tukey’s test). **(D)** Western blot analysis of pro-inflammatory cytokines in the rat lung. **(E)** Expression of TNF-α in lung tissue (post-hoc analysis: Tukey’s test). **(F)** Expression of IL-6 in lung tissue (post-hoc analysis: Tukey’s test). **(G)** Expression of IL-1β in lung tissue (post-hoc analysis: Tukey’s test). Data were expressed as mean ± SEM (*n* = 7), *p < 0.05, **p < 0.01, ***p < 0.001 vs. *SHAM group*; ^*#*^ *p <* 0.05, ^*##*^ *p <* 0.01, ^#*##*^ *p <* 0.001 vs. *IR group*.

As shown in Figure 7, in the *IR group* and in the *IR + ACE2-inhibited group*, there was increased expression of p38MAPK and p65NF-κB during lung IR injury (level of p38MAPK *SHAM* vs. *IR group*, *p < 0.01, SHAM* vs. *IR + ACE2-inhibited group, p = 0.064*; level of p65NF-κB *SHAM* vs. *IR group*, *p < 0.01, SHAM* vs. *IR + ACE2-inhibited group, p < 0.01*). Furthermore, there was increased expression of pro-inflammatory cytokines TNF-α, IL-6, and IL-1β (level of TNF-α, *SHAM* vs. *IR group*, *SHAM* vs. *IR + ACE2-inhibited group, p <* 0.05, respectively; level of IL-6, *SHAM* vs. *IR group*, *SHAM* vs. *IR + ACE2-inhibited group*, *p <* 0.001, respectively; level of IL-1β, *SHAM* vs. *IR group*, *SHAM* vs. *IR + ACE2-inhibited group*, *p <* 0.01, respectively). Treatment with DIZE was associated with downregulation of p65NF-κB (level of p65NF-κB *IR + DIZE* vs. *IR group, p < 0.05*) and a decreasing trend of p38MAPK expression, however, without a statistical difference (level of p38MAPK *IR + DIZE* vs. *IR group, p = 0.077*). Additionally, expressions of IL-6 and IL-1β were decreased in the *IR + DIZE group* (level of IL-6, *IR + DIZE* vs. *IR group*, *p < 0.001*; level of IL-1β, *IR + DIZE* vs. *IR group*, *p < 0.01*).

### DIZE Protected Human Pulmonary Epithelial Cells Against OGD/R Injury

**FIGURE 8:**
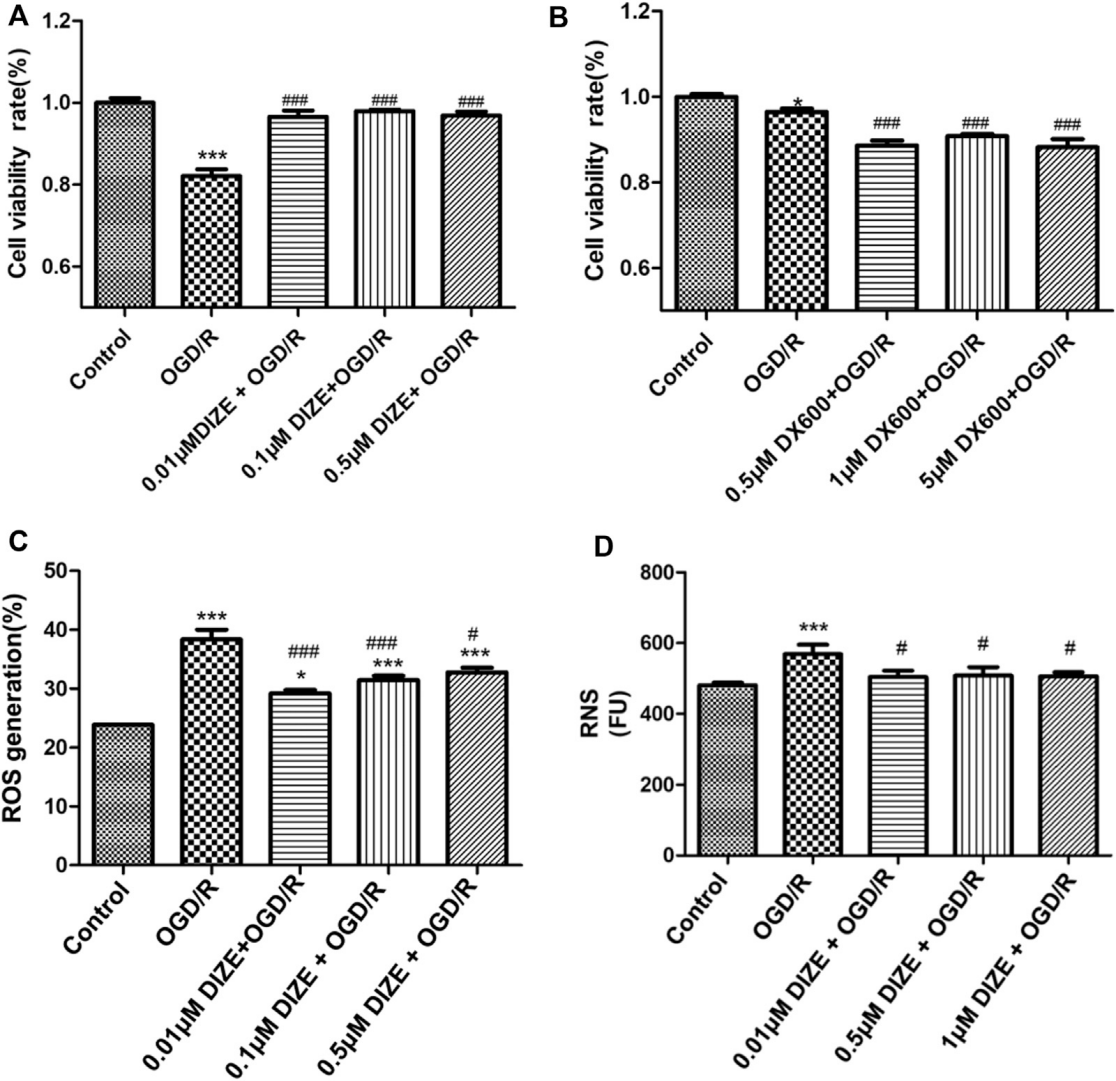
DIZE protected human pulmonary epithelial cell against OGD/R injury. **(A)** Cell viability was measured via CCK-8 assay. A549 cells were pre-treated with DIZE (0.01, 0.1, 0.5 μM) for 24 h, and exposed to oxygen-glucose deprivation (OGD) for 8 h, followed by reperfusion period for 12 h **(B)** DX600 was a potent ACE2-specific inhibitor. A549 cells were pre-treated with DX600 (0.5, 1, 5 μM) for 24 h, and exposed to OGD for 8 h, followed by reperfusion period for 12 h. ACE2 inhibition could worsen OGD/R injury to human pulmonary epithelial cells. **(C)** DIZE alleviated intracellular reactive oxygen species (ROS) production during OGD/R injury in A549. **(D)** DIZE alleviated intracellular reactive nitrogen species (RNS) production during OGD/R injury in A549. *p < 0.05, ***p < 0.001 vs. *Control group*; ^#^ *p <* 0.05, ^##^ *p <* 0.01, ^###^ *p <* 0.001 vs. *OGD/R group*.

*In vitro*, the OGD/R model was set at oxygen–glucose deprivation of 8 h, followed by 12 h of reperfusion in A549 cells, which could cause significant cell damage and thus simulated lung IR injury (cell viability rate *Control* vs. *OGD/R group*, *p <* 0.001). Pretreatment with DIZE significantly enhanced cell survival, protecting cells against OGD/R injury (Figure 8A) (cell viability rate *OGD/R* vs. *OGD/R+0.01 μM DIZE*, *OGD/R* vs. *OGD/R+0.1 μM DIZE*, *OGD/R* vs. *OGD/R+0.5 μM DIZE*, *p <* 0.001, respectively). Contrastingly, inhibition of ACE2 with DX600 significantly aggravated cell injury due to OGD/R (Figure 8B) (cell viability rate *OGD/R* vs. *OGD/R+0.5 μM DX600*, *OGD/R* vs. *OGD/R+1 μM DX600*, *OGD/R* vs. *OGD/R+5 μM DX600*, *p <* 0.001, respectively). Additionally, A549 cells in the OGD/R model exhibited increased indicators of both oxidative and nitride stress (ROS generation, *Control* vs. *OGD/R group*, *p <* 0.001; RNS generation, *Control* vs. *OGD/R group*, *p* < 0.001), whereas DIZE effectively reduced intracellular production of ROS and RNS, thus downregulating the production of a series of toxic cytokines (ROS generation, *OGD/R* vs. *DIZE + OGD/R groups*, *p <* 0.05 respectively; RNS generation, *OGD/R* vs. *DX600+OGD/R groups*, *p <* 0.05 respectively; Figures 8C,D).

### DIZE Pretreatment Protected ACE2 Enzyme Activity During OGD/R Injury

**FIGURE 9:**
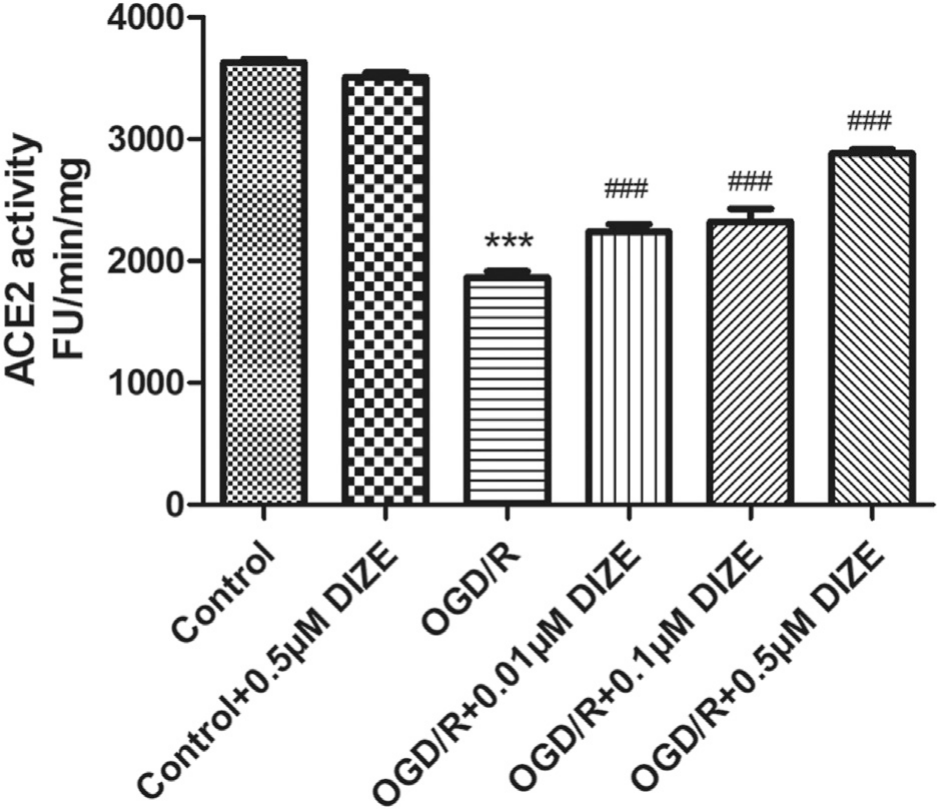
DIZE protected ACE2 enzyme activity during OGD/R injury. OGD/R injury significantly decreased ACE2 activity in A549. DIZE pretreatment (0.01, 0.1, 0.5 μM) preserved higher ACE2 enzyme activity during OGD/R injury in A549. ***p < 0.001 vs. *Control group*; ^*###*^ *p* < 0.001 vs. *OGD/R group*.

Fluorescence spectroscopy was applied to determine the enzyme activity of ACE2. As shown in Figure 9, compared to that of the *Control group*, relative enzyme activity of ACE2 significantly decreased under the conditions of OGD/R (*p <* 0.001, vs. *Control group*). However, DIZE pretreatment protected the enzyme activity of endogenous ACE2 (*p <* 0.001, vs. *OGD/R group*).

## Discussion

This is the first study to show that the protection of endogenous ACE2 could ameliorate lung reperfusion injury both *in vivo* and *in vitro*, which may have profound clinical implications in the future. ACE2 protectant DIZE could inhibit ADAM17-mediated ACE2 shedding, thus preserving endogenous ACE2 enzyme function in the lung tissue and significantly reducing pathophysiological changes during lung injury.

We have used both animal and human pulmonary epithelial cell IR injury models resembling surgical scenarios for the lung reperfusion period during pulmonary endarterectomy or lung transplantation. The NHLBI/NIH have indicated that left hilum occlusion followed by reperfusion in rats is a well-established *in vivo* model, mimicking warm ischemia period during human lung transplantation (Lama et al., 2017). Jia et al. (2014) estimated that the duration of the survival time of the IR mice model was 30–60 min after reperfusion. Therefore, we checked all the physiological parameters 45 min after reperfusion in our rat model as a reasonable endpoint for observation. There were no perioperative deaths recorded in our study. In our model, lung deflation during both ischemia and re-expansion during reperfusion phase, along with hemodynamic fluctuation, increased W/D and decreased OI; all testified to proper animal model establishment (Ozbek et al., 2009; Matute-Bello et al., 2011; Gielis et al., 2015).

**FIGURE 10:**
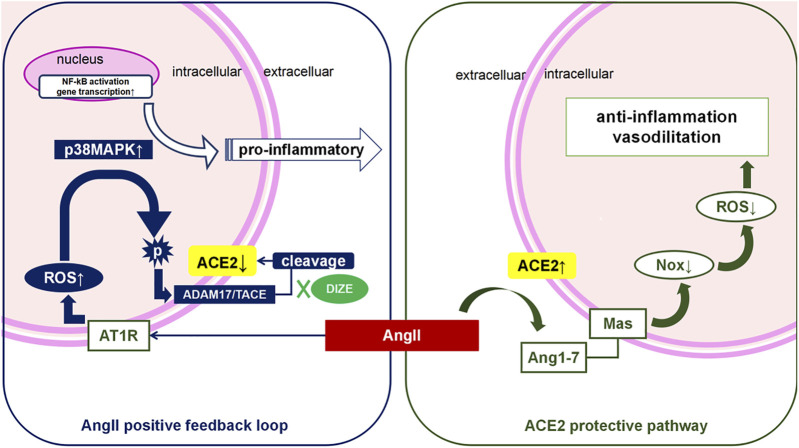
AngII positive feedback loop and ACE2 protective pathway during lung IR injury. During lung IR injury, hypoxia injury triggers Angiotensin II (AngII) secretion from epithelial, endothelial and pulmonary smooth muscle cells, which is worsened by oxidative stress during the reperfusion period (“the double whammy”). Activation of the AngII-AT_1_R pathway further triggers the reactive oxygen species (ROS) cascade. Meanwhile, there is a homeostatic mechanism that causes excessive AngII to be degragated via ACE2 on the cell membrane, which counterbalances the function of AngII. However, persistent high level of AngII, overload oxidative stress, or other pro-inflammatory factors could all activate phosphorylation of ADAM17, and phospho-ADAM17 cleaves the extracellular functional domain of ACE2, thus deactivating ACE2. The “positive AngII feedback loop” results in acute or sub-acute lung reperfusion injury. We have demonstrated that DIZE pretreatment is associated with lower levels of ADAM17, and that DIZE could suppress the phosphorylation of ADAM17, thus inhibiting ADAM17-mediated ACE2 shedding. Protection of endogenous ACE2 enzyme function during lung IR injury is associated with the reduction of both oxidative stress and nitrosative stress, and decreased inflammatory biomarkers, leading to significant improvement of respiratory function and lung pathology.

Although the underlying mechanism of lung IR injury remains unclear, previously proposed pathophysiological mechanisms of lung IR injury include hypoxic injury and accumulation of toxic metabolites due to ischemic hypoxia and oxidative stress and pro-inflammatory cascade during reperfusion. Both of these suggest a “double whammy” effect on the respiratory membrane, resulting in diffuse pulmonary edema and pulmonary vasospasm. Ventilation/blood flow ratio disorder and pulmonary hypertension are identical clinical manifestations of lung IR injury. As shown in Figure 10, the balance of the ACE-AngII-AT_1_R/ACE2-Ang1-7-Mas system depends on both positive and negative feedback. Research evidence suggests that the degradation of over expressed AngII via ACE2 is the key protective pathway against an injurious environment for lungs. Based on previous studies, we speculated that ACE2 would be an innovative detection and intervention target throughout the whole process of lung IR injury. Supporting evidence from previous studies includes the following: 1) For the ischemic hypoxic stage, the most immediate hypoxia-dependent responses include IL-1β-mediated NO release through MAPK and NF-κB pathway (Park et al., 2001; Kim et al., 2006). Jugdutt (2002) reported that in a myocardial IR model, eNOS dysfunction was AngII dependent. In contrast, Li et al. (2018) also found that supplementation of ACE2 could maintain NO-induced vasodilatory effects in a model of pulmonary hypertension, thus decreasing pulmonary artery pressure. 2) During the reperfusion phase, the typical damage caused by IR injury includes oxygen free radicals and an oxidative-inflammatory cascade that leads to endothelial dysfunction and decreased NO release. As for the oxidative stress pathway, Zhang et al. (2018); Thatcher, Gupte, Hatch, Cassis) reported that excessive AngII could reduce endogenous ACE2 in endothelial cells, and AngII could induce overproduction of ROS while reducing NO production. Conversely, ACE2 upregulation in endothelial cells could increase the production of NO, thus protecting endothelial cells from oxidative stress induced by AngII. TNF-α is the initiator cytokine of the pro-inflammatory cascade. As such, a deficiency of ACE2 in macrophages could increase TNF-α expression and promote an inflammatory micro-environment (Jugdutt, 2002). In contrast, enhancement of ACE2 activity could prevent TNF-α stimulated ICAM-1 expression and phosphorylation of NF-κB, indicating that ACE2 has an anti-inflammatory feature (Zhu et al., 2015). Therefore, we speculated that the enzyme function of ACE2 was the key factor maintaining the pulmonary micro-environment against lung IR injury.

Further, as previously discussed, we testified that lung IR injury exhibited a significant increase of pro-inflammatory cytokines TNF-α, IL-6, and IL-1β, as well as activation of MAPK/NF-κB-signaling pathway, which was responsible for the initiation of the inflammatory cascade. Pretreatment of DIZE during lung IR injury significantly decreased NF-κB, IL-6, and IL-1β levels. There was a trend but without statistically significantly reduced p38MAPK (*p =* 0.077) and TNF-α (*p >* 0.1). However, as previously discussed, MAPK/NF-κB/TNF-α initiates a series of pro-inflammatory cytokine expression. Treatment of DIZE inhibited levels of nonspecific inflammatory factors, consistent with pathological manifestations of its protective effect to the lung structure, decrease of the index of pulmonary edema W/D ratio, as well as the improvement of oxygenation index during lung IR injury. The possible protective effects of the protection of ACE2 function during the early stage of lung IR injury were illustrated from multiple perspectives in our experiment. Together with previous literature, our study supported that DIZE maintaining endogenous ACE2 activity could reduce MAPK-NF-κB inflammatory pathway, thus downregulated the level of pro-inflammatory cytokines in rat lung.

It is consistent with previous findings on human lung tissue (He et al., 2006; Baker et al., 2021), there was abundant distribution of ACE2 in the pneumocytes and broncial tissue in rat lung. In our study, it was demonstrated that lung IR injury was associated with both pulmonary ACE2 dysfunction and upregulation of the AngII-AT_1_R pathway. Both the *in vivo* and *in vitro* model showed an overexpression of AngII and AT_1_R during lung IR injury, reduced levels of the membrane bound form of ACE2, as well as weakened ACE2 enzyme activity during lung IR injury. In addition, we found that specific suppression of ACE2 in both models directly increased the degree of lung IR injury.

A decrease in ACE2 could be exacerbated by the compounds MLN-4760 and DX600, the only two selective ACE2 inhibitors in the market. DX600 only inhibits the enzyme activity of ACE2 in human tissue. Moreover, MLN-4760 is a potent and selective human ACE2 inhibitor that has been reported to have some effect on murine organs (Ye et al., 2012). A more precise method of ACE2 inhibition might be the *ACE2* gene knockout mice model. Due to economic concerns and difficulties operating on mice to build the lung IR injury model, it was not used in this experiment. However, we found that the ACE2 inhibitor given to the rats did not work best to aggravate the imbalance of AngII/ACE2 during lung IR injury. As shown in our results, in the *ACE2-inhibited group*, some rats exhibited severe alveolitis as a pathological manifestation (Figures 4C,D), characterized by massive pink fluid exudates in the alveolar cavities. However, lung injury score (LIS), W/D ratio, and molecular biological markers seemed to exhibit some trends of severe lung injury but did not indicate statistical differences between the *IR + ACE2-inhibited group* and *IR group*. In contrast, when evaluating the therapeutic effect of DIZE pretreatment, comparing between *IR + ACE2-inhibited group* and *IR + DIZE group*, during lung IR injury, DIZE could significantly reduce the accumulation of excessive AngII-AT_1_R and could reduce the loss of endogenous ACE2 in lung tissue, as well as reducing pro-inflammatory cytokines such as NF-κB, IL-6, and IL-1β. Additionally, we compensated for this in our complementary cell experiment. In an *in vitro* experiment, we used DX600, a selective human ACE2 inhibitor, on human alveolar epithelial cells. In OGD/R model, DX600 significantly reduced cell survival, whereas treatment with DIZE could increase the enzyme activity of ACE2 and improve cell survival rate in the OGD/R model. Our study further demonstrated that inhibition of ACE2 enzyme activity could deteriorate the degree of pulmonary reperfusion injury and aggravate the alveolar epithelial injury. And the protection of endogenous ACE2 enzyme activity could reduce the degree of lung IR injury, thus exhibited a protective role in the human alveolar epithelium.

A promising finding was that the DIZE pretreatment group in lung IR injury animals achieved higher OI as well (OI increased from 333 to 459 mmHg) and reduction of pulmonary edema as comprehensive respiratory function indicators. We performed further tests to clarify the mechanism of DIZE on ACE2 enzyme activity during lung IR injury. It has been reported that the deactivation mechanism for ACE2 activity is due to ectodomain shedding via ADAM17 (Xu et al., 2018). AngII-AT_1_R activation results in the upregulation of ACE2 expression; however, further, it could promote ADAM17 phosphorylation (53). Thus, the activation of ADAM17 could be triggered by excessive AngII, oxidative stress, and MAPK pathway (Scott et al., 2011; Xu et al., 2017). ADAM17 cleaves the extracellular N-terminal active site of ACE2, shedding the ectodomain of ACE2 from the cell membrane. ADAM17-mediated ACE2 shedding would result in increased ACE2 fragments and decreased physiologically active ACE2 in local tissue (Ye et al., 2012), consequently helping to reinforce the vasoconstrictive and pro-inflammatory effect of AngII. It was known that upregulation of ADAM17 was responsible for ACE2 shedding, thus impairing the role of ACE2 in pathophysiological activities (Lama et al., 2017). In our study, the inhibition of endogenous ACE2 during IR injury was accompanied by increased ADAM17. Furthermore, DIZE did not alter the enzyme activity of ACE2 under physiological conditions. However, under the condition of lung IR injury, DIZE significantly reduced the level of ADAM17 and maintained a relatively higher level of ACE2. Our study strongly indicated that DIZE could inhibit ADAM17-mediated ACE2 shedding, thus possibly preserving endogenous ACE2. Moreover, DIZE ameliorated IR injury in both animal model and in human alveolar epithelial cells, which might indicate a possible therapeutic effect on lung IR injury-induced hypoxemia, lung edema, and pulmonary pathological manifestations.

DIZE is an anti-Trypanosoma cruzi agent for animal use only, and is not licensed for humans (Kuriakose et al., 2012). Moreover, its effect on ACE2 had not been investigated until the beginning of the 21st century. Thus, its off-target treatment effect remains unclear. To our knowledge, only one study published in 1968 reported that oral and intravenous DIZE administration was safe for humans, and that its use might have a preventive effect to trypanosomosis. Nevertheless, the authors did not report on the applied dose (Bailey, 1968). Therefore, there is no practical reference for the drug safety. Current evidence for human use of DIZE remains limited to *in vitro* cell experiments. Concerning its clinical application, maintaining endogenous ACE2 might have an effect on systemic blood pressure under the circumstance of IR injury. Its safety on humans and whether it would cause cardiovascular adverse events remain to be verified. In the follow-up study, we hope to develop a novel tool drug modulating endogenous ACE2 shedding in the treatment of lung IR injury and to achieve the possible application of the ACE2 shedding mechanism for early detection of lung IR injury.

## Conclusion

In this study, we demonstrated that at early-stage lung IR injury, AngII/ACE2 exhibited significant imbalance. Lung IR injury was marked by impaired ACE2 activity and increased levels of AngII in lung tissue. We were the first to apply DIZE in a rat lung IR injury model and found that DIZE could preserve endogenous ACE2 function, alleviating pathophysiological processes during lung IR injury. Furthermore, DIZE could decrease ADAM17 levels during lung injury, thus inhibiting ADAM17 associated ACE2 shedding. Preservation of endogenous ACE2 was associated with decreased oxidative and nitrosative stress pathways, thus resulting in better oxygenation and better respiratory and circulatory function.

## Data Availability

The original contributions presented in the study are included in the article/Supplementary Material, further inquiries can be directed to the corresponding author.
